# Neutrophil predominance in bronchoalveolar lavage fluid is associated with disease severity and progression of HRCT findings in pulmonary *Mycobacterium avium* infection

**DOI:** 10.1371/journal.pone.0190189

**Published:** 2018-02-05

**Authors:** Takashi Inomata, Satoshi Konno, Katsura Nagai, Masaru Suzuki, Masaharu Nishimura

**Affiliations:** First Department of Medicine, Hokkaido University School of Medicine, Sapporo, Japan; Rutgers Biomedical and Health Sciences, UNITED STATES

## Abstract

Pulmonary *Mycobacterium avium* complex (MAC) infection is increasing in prevalence worldwide even in immunocompetent individuals. Despite its variable clinical course, the clinical and immunological factors associated with radiographical severity and progression are not largely unknown. We aimed to study the association between the inflammatory cell and cytokine profiles at the local infected site, and the radiological severity and/or progression of pulmonary MAC infection. In this retrospective cohort study, 22 healthy subjects and 37 consecutive patients who were diagnosed as having pulmonary MAC infection by positive cultures of bronchoalveolar lavage (BAL) fluids were enrolled. The 37 patients were divided into 2 groups based on the predominant BAL inflammatory cell type: the lymphocyte-dominant (LD) group and neutrophil-dominant (ND) groups. The high-resolution computed tomography score in both the lavaged segment and whole lung and cytokines profiles were compared between the 2 groups. The clinical course after the BAL procedure was also compared between the 2 groups. Both the segment and whole lung scores in the ND group were significantly higher than the LD group (P < 0.001). Levels of IL-8 in the BAL fluids were significantly higher in the ND group compared to the LD group (P = 0.01). In contrast, levels of IL-22 were significantly lower in the ND group compared to the LD group (P < 0.001). The prevalence of patients who showed deterioration of the disease was significantly higher in the ND group (83.3%) than the LD group (12.5%) (P < 0.01).

Neutrophil-predominant inflammatory response at the infected site is associated with the radiographical severity and progression of pulmonary MAC infection.

## Introduction

Chronic pulmonary infection with *Mycobacterium avium* (*M*. *avium*) and *M*. *intracellulare*, referred to as the *M*. *avium* complex (MAC), is being recognized worldwide with increasing frequency even in immunocompetent patients [1–3]. Pulmonary MAC infection can lead to a range of clinical outcomes [4–8]: Some patients demonstrate stable radiographic findings with minimal symptoms for a relatively long period, whereas others have a progressive disease leading to morbidity and mortality even with the use of antibiotics. However, to date, the factors associated with disease severity and/or progression of the disease are not completely understood.

Chest CT imaging is useful for evaluating the severity and expansion of pulmonary MAC infections and also for differentiating between different types of the disease (i.e., nodular or fibro-cavitary type). Clinicians utilize CT imaging, along with patients’ symptoms, as a guide to initiate chemotherapy. In addition, some previous studies objectively evaluated the scoring of each item from CT imaging and evaluated the severity and deterioration of pulmonary tuberculosis and MAC infection [6,8–12].

Increased neutrophil and lymphocyte counts are characteristic of the bronchoalveolar lavage fluid (BALF) cellular constituents in patients with pulmonary *Mycobacterium* infection, including *M*. *tuberculosis* and MAC [6,13–17]. In previous studies, the potential roles of neutrophils in causing disease progression and that of lymphocytes in protection were suggested [13–17]. Based on the BALF findings, Yamazaki et al. reported that an increase in the percentage of neutrophils and a reciprocal decrease in the lymphocytes were associated with a deterioration in patients with pulmonary MAC infection [6]. However, in this study, the authors did not examine the details of the association between the cell differentiation status and disease severity in the lavaged segment, which could have been evaluated by high-resolution computed tomography (HRCT). Further, there have been no studies that biologically characterized patients with MAC infection based on the cell differentiation status and cytokine profiles in the BALF.

In most cases of pulmonary MAC infection, a definite diagnosis is established by positive culture results from at least two separate expectorated sputum samples. However, when sputum cannot be obtained or when sputum cultures fail to demonstrate the presence of bacteria, diagnostic BAL can be performed for a suspected lobe. In the present study, we focused on subjects who performed BAL by reasons above and made a diagnosis of MAC, we analyzed the correlation between the specific HRCT findings in both the lavaged segments and the whole lung, and the local cellular immunological status which was evaluated the cell differentiation in BALF. Given the potential contrasting roles of neutrophils and lymphocytes in the development of pulmonary *Mycobacterium* infection as described in previous reports [13–17], we specifically hypothesized that a predominant immune response by either lymphocytes or neutrophils might determine the severity and/or progression of pulmonary MAC infection. For gaining insights into the underlying mechanism(s) of the disease at a molecular level, the levels of several cytokines and/or chemokines in the BALF were measured. Furthermore, for more clinical relevance, we selected patients who were followed-up after the BAL procedure without any treatment, and retrospectively evaluated whether the cell and cytokine profiles and HRCT findings were associated with disease progression.

## Materials and methods

All patients provided their written informed consent for participating in this study and for their samples to be used in future clinical research. The Institutional Review Board of Hokkaido University Hospital for Clinical Research approved the study protocols (approval number 010–0337).

### Study subjects

A total of 37 patients with pulmonary MAC infection were enrolled in this retrospective cohort study. They had been previously diagnosed using BAL at the First Department of Medicine, Hokkaido University Hospital, between April 2000 and March 2014. In all patients, pulmonary MAC infection was initially suspected on the basis of a clinical history of cough and sputum and HRCT findings of patchy areas of bronchiectasis and branching centrilobular nodules (i.e., tree-in-bud pattern). As the sputum cultures failed to demonstrate MAC infection several times, diagnostic BAL was performed for a suspected lobe. The diagnosis of pulmonary MAC infection was confirmed according to the criteria of the American Thoracic Society / Infectious Disease Society of America [18] in all patients. Patients with positive cultures other than MAC, such as other bacteria and other species of non-tuberculous mycobacteria (NTM) in BALF, were excluded. Patients with pulmonary MAC infection with fibro-cavitary presentation on HRCT were also excluded. Patients who had other concomitant pulmonary diseases, such as chronic obstructive pulmonary disease (COPD), lung cancer, tuberculosis and other pulmonary infections, idiopathic/secondary interstitial pneumonia, and sarcoidosis, were also excluded. All patients were considered immunocompetent; thus, they did not have acquired immune deficiency syndrome, autoimmune diseases, hematopoietic diseases, administration of immunosuppressive medication, or any kind of malignant diseases. Twenty-two healthy volunteers (7 men and 15 women; median age, 60 years; range, 20–71 years) with no history of any pulmonary diseases or respiratory symptoms were recruited as controls for the study and underwent BAL. All subjects provided their written, informed consent for participating in this study and for their samples to be used in future clinical research.

### BAL and blood analysis

The patients underwent BAL, as described previously in the reports from our laboratory [19,20]. Briefly, the affected segmental bronchus was identified on the chest computed tomography (CT) scan, and was lavaged 3 times using 50-mL aliquots (total volume, 150 mL) of sterile 0.9% saline at room temperature through a wedged flexible fiberoptic bronchoscope (Olympus 1T-200 or 1T-240, Tokyo, Japan). The BALF obtained was separately filtered through several layers of gauze to remove excess mucus and debris, and was subsequently centrifuged at 1500 rpm for 5 min at 4°C to separate the supernatant from the cells. Aliquots of the supernatant (1 mL) were immediately frozen and stored at -80°C prior to the assay. Cell pellets were counted in a hemocytometer, and Diff-Quik^™^ (International Reagents, Kobe, Japan)-stained smears were used to identify the differential profiles after cytospin preparation. Differential counts were performed by examining 300 cells using a standard light microscope. The T lymphocyte subpopulations were determined using flow cytometry. Whole blood samples were incubated with fluorescent conjugated monoclonal antibodies CD3, CD4, and CD8 (Beckman Coulter, Tokyo, Japan). The BAL samples were incubated with fluorescent monoclonal antibodies CD3, CD4, CD8, and CD45 (Beckman Coulter). The samples were subsequently stained at room temperature in the dark for 15 min. Red blood cell (in whole blood samples) lysis was performed using the Q-Prep lysing kit (Beckman Coulter). Cells were then washed and resuspended in PBS. Cells were analyzed with a flow cytometer (Cytomics FC500, Beckman Coulter, Tokyo, Japan). Venous blood was withdrawn 30 minutes before the BAL. After collection, serum samples were immediately frozen and stored at -80°C until the assay.

### Chest CT scan and scoring

Thin-slice (1-mm) CT images were obtained at 10-mm intervals from the lung apices to the bases during suspended full inspiration in the supine position, within 1 month prior to the BAL. All CT data were reconstructed using a high spatial frequency algorithm and displayed on the lung parenchymal window (level, -700 Hounsfield units (HU); width, 1200 HU). The HRCT images were scored for the severity and extent of pulmonary MAC infection using a modified scoring system by Fowler et al. [9], for both the lavaged segment and whole lungs, as briefly summarized in Table 1 and S1 Table. The presence of bronchiectasis was determined using established criteria [18]. The severity of bronchial dilatation was evaluated on the basis of previously described criteria [11] (0, absent; 1, the luminal diameter was slightly greater than but did not exceed twice the diameter of the adjacent vessel; 2, the luminal diameter was 2–3 times the diameter of the adjacent vessel; and 3, the luminal diameter exceeded 3 times the diameter of the adjacent vessel). The bronchial wall thickening was scored for each segment using the criteria in the paper above [9] (0, absent; 1, the bronchial wall thickness was less than one half the diameter of the adjacent vessel; 2, the bronchial wall thickness was greater than one half, but did not exceed the diameter of the adjacent vessel; and 3, the bronchial wall thickness was greater than the diameter of the adjacent vessel). In cases where bronchiectasis and bronchial wall thickening were not of uniform severity throughout the segments and the lungs, scoring was based on the most frequently identified severity. The extent of bronchiectasis, multiple nodules or small nodules, sacculations/abscesses, mosaic perfusion, and collapse/consolidation were assessed segment-wise [11,12]. The number (0, none; 1, 1–5 segments; 2, 6–9 segments; and 3, >9 segments) and the proportion (0, absent; 1, <25%; 2, 25–50%; and 3, >50%) of segments involved were scored in whole lung analysis and lavaged-segment analysis, respectively. The lavaged-segment and whole lung score for all CT features were derived by summing the scores for the individual CT features. Chest CT scans were evaluated simultaneously by 2 respiratory physicians (TI and SK) in a random order without knowledge of the patient’s clinical course. Final conclusions regarding the CT scan findings were arrived at by a consensus. S1 Fig shows the representative HRCT images. S3 and S4 Tables show the kappa values for each item between the 2 physicians; kappa ranged from 0.466 to 1.00.

**Table 1:** Summary of the HRCT scoring system of the lavaged lung.

Category	Score			
	0	1	2	3
Severity of Bronchiectasis	Absent	Mild (1–2 x diameter of adjacent vessel)	Moderate (2–3 x diameter of adjacent vessel)	Severe (>3 x diameter of adjacent vessel)
Severity of bronchial wall thickening	Absent	Mild (<0.5 x diameter of adjacent vessel)	Moderate (0.5–1 x diameter of adjacent vessel)	Severe (>1 x diameter of adjacent vessel)
Extent of bronchiectasis (% of segmental area)	Absent	1%-25%	25%-50%	>50%
Extent of multiple nodules or small nodules (% of segmental area)	Absent	1%-25%	25%-50%	>50%
Sacculations or abscesses (% of segmental area)	Absent	1%-25%	25%-50%	>50%
Extent of mosaic perfusion (% of segmental area)	Absent	1%-25%	25%-50%	>50%
Collapse or consolidation (% of segmental area)	Absent	1%-25%	25%-50%	>50%

HRCT; high-resolution computed tomography

### Measurement of cytokine levels in the BALF

Cytokine levels in the BALF were measured using the following enzyme-linked immunosorbent assay (ELISA) kits: interleukin (IL)-8, IL-12p70, IL-17A, IL-22, interferon (IFN)-γ (eBioscience Inc., San Diego, CA, USA), IL-6 (R&D Systems, Minneapolis, MN, USA), and IL-18 (MBL, Nagoya, Japan). The sensitivity of these assays was 4 pg/mL for IFN-γ, 0.1 pg/mL for IL-12, 0.7 pg/mL for IL-6, 2 pg/mL for IL-8, 8 pg/mL for IL-22, 12.5 pg/mL for IL-18, and 30 pg/mL for IL-17A. Undetectable values were assigned an arbitrary value of half the sensitivity limit.

### Follow-up of HRCT findings

Of the 37 patients, 6 were initiated on chemotherapy immediately after the confirmation of MAC infection by BAL. Three patients were sent back to referral hospitals and 6 were lost to follow-up. Thus, 22 patients were retrospectively available for evaluation the change in HRCT findings after the BAL procedure without initiation of treatment.

### Statistical analysis

For analyses, patients (N = 37) were divided into 2 groups based on the predominant percentage of the inflammatory cell type.

1. Lymphocyte-predominant (LD) group: patients with a higher proportion of lymphocytes (%) than neutrophils (%) in the BALF (N = 22)
2. Neutrophil-predominant group (ND): patients with a higher proportion of neutrophils (%) than lymphocytes (%) in the BALF (N = 15)

For evaluating the longitudinal clinical course without any treatment for MAC infection, the patients (N = 22) were divided into 2 groups.

1. Stable group (N = 15): patients whose HRCT findings did not worsen at one-year follow-up after the BAL procedure
2. Deteriorated group (N = 7): patients who showed worsening of HRCT findings, such as nodules, bronchiectasis, and/or consolidation or/and the advent of new lesions suspicious of MAC infection in other lobe(s) at least 3 months after the BAL procedure without any treatment (mean observation period, 9.6 months; range, 3–12 months)

The HRCT findings were reviewed independently by 1 radiologist and 2 respiratory physicians (TI and SK). No disagreements among the 3 doctors were observed for any of the subjects.

Statistical analyses were performed using the statistical software package SYSTAT for Windows, version 13.1 (SYSTAT, San Jose, CA, USA). The results were expressed as mean ± SEM. The Mann-Whitney U test was applied for statistical evaluation of differences between the 2 groups. Comparisons of proportions were made using the Fisher’s exact test. Changes in CT scores before and after the follow-up were assessed using the Wilcoxon signed-rank test. Interobserver variation for the CT scores was assessed using Cohen’s kappa coefficient of agreement. A P-value of less than 0.05 was regarded as statistically significant.

## Results

### Cell profiles in the BALF (Fig 1)

**Fig 1:**
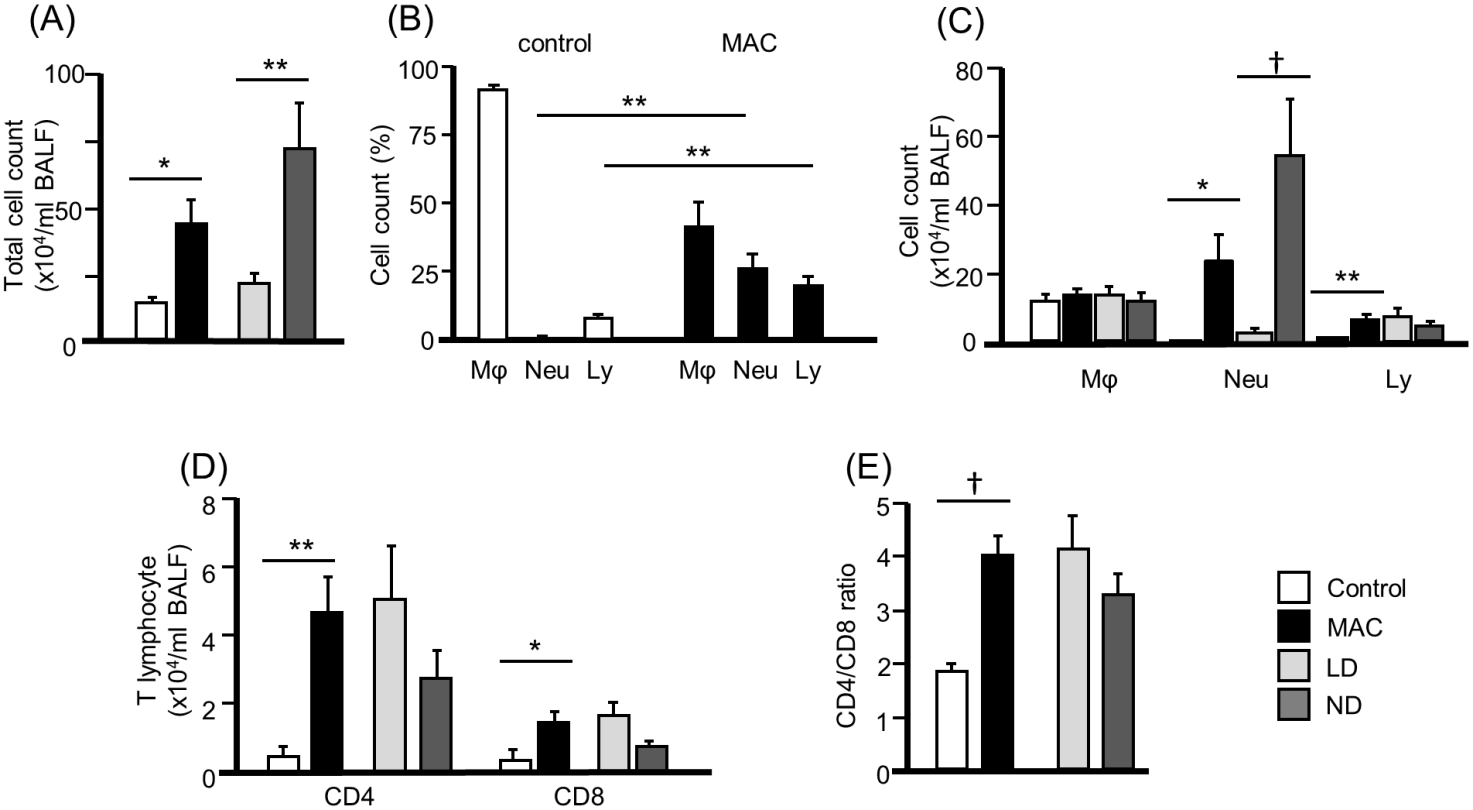
Total, differential cell and lymphocyte profiles in the BALF of control and MAC patients. Cell pellets were counted in a hemocytometer, and Diff-Quik^™^ (International Reagents, Kobe, Japan)-stained smears were used to identify the differential profiles after cytospin preparation. Differential counts were performed by examining 300 cells using a standard light microscope. Data are represented as means ± SEM. *P < 0.05 ** P < 0.01 †P < 0.001. Mϕ = Macrophage, Neu = Neutrophil, Ly = Lymphocyte. LD: lymphocyte-dominant. ND: neutrophil-dominant. MAC: *Mycobacterium avium* complex.

The total count of cells in the BALF of patients with pulmonary MAC infection (41.3 x 10^4^/mL) was significantly higher than control subjects (12.7 x 10^4^/mL) (P < 0.05) (Fig 1A). The mean numbers and percentages of neutrophils (25.9%) and lymphocytes (19.9%) in the MAC group were significantly higher than the control group (P < 0.05) (Fig 1B and 1C). In the analysis of lymphocytes subsets, the numbers of CD4+ and CD8+ T lymphocytes in the BALF of the MAC group (4.1 x 10^4^/mL and 1.3 x 10^4^/mL, respectively) were significantly higher than the control group (P < 0.05, respectively) (Fig 1D). The mean CD4/CD8 ratio of the MAC group (3.8) was significantly higher than the control group (1.8) (P < 0.001) (Fig 1E).

### Characteristics of patients

Of the 37 patients, 22 were categorized into the lymphocyte-dominant (LD) group and 15 into the neutrophil-dominant (ND) group. The baseline characteristics of healthy controls and patients are shown in Table 2 and S1 File. Gender and smoking status were similar between healthy controls and patients. The mean age of the patients was significantly higher than that of the healthy patients. There were no significant differences between the LD and ND groups in terms of age, sex, and smoking history.

**Table 2:** Subject characteristics.

Characteristics	Control (N = 22)	MAC patients			P value	
		All (N = 37)	LD (N = 22)	ND (N = 15)	Control vs. MAC	LD vs. ND
Age(y), median[min-max]	60[20–71]	65[45–78]	66[45–78]	62[47–76]	0.03	0.60
Sex (M/F)	7/15	4/33	2/20	2/13	0.08	0.90
Cigarette smoking status						
Current/Former/Never	1/4/17	1/7/29	0/3/19	1/4/10	0.90	0.23
MAC strain						
*M*. *avium*		33	20	13		0.90
*M*. *intracellulare*		4	2	2		

Data are presented as the number of subjects except median age. LD; Lymphocyte-dominant group, ND; Neutrophil-dominant group.

Table 3 and Fig 1 show the comparisons of the cellular responses between the LD and ND groups. The total cell counts in the ND group (70.8 x 10^4^/mL) were significantly higher than in the LD group (21.2 x 10^4^/mL) (P < 0.01) (Fig 1B). The neutrophil count in the ND group (54.8 x 10^4^/mL) was significantly higher than the LD group (0.8 x 10^4^/mL) (P < 0.001). The number of lymphocytes was not different between the two groups [LD group (7.0 x 10^4^/mL), ND group (4.0 x 10^4^/mL)] (P = 0.30). The counts of macrophages were not different between the 2 groups. The number of peripheral white blood cells in the MAC patients (5208 x 10^4^/mL) was not significantly different compared to the control group (5506 x 10^4^/mL) (S2 Table). The counts of peripheral neutrophils and lymphocytes were not different between the groups; eosinophil and monocyte counts showed significant differences. There was no significant difference in the peripheral white blood cell counts and its cell differentiation between the LD and ND group (S2 Table).

**Table 3:** Cell differentiation status in the lungs of different groups.

	Control (N = 22)	MAC patients			P value	
		All (N = 37)	LD (N = 22)	ND (N = 15)	Control vs. MAC	LD vs. ND
Total cell count (x10^4^/ml)	12.7 ± 1.8	41.3 ± 8.5	21.2 ± 4.3	70.8 ± 17.7	0.013	0.003
Neutrophils (%)	0.7 ± 0.2	25.9 ± 5.6	3.3 ± 0.8	58.9 ± 8.0	0.001	<0.001
Neutrophils (x10^4^/ml)	0.1 ± 0.1	22.7 ± 8.0	0.8 ± 0.2	54.8 ±16.9	0.03	<0.001
Lymphocytes (%)	7.6 ± 1.4	19.9 ± 3.1	28.8 ± 4.1	6.8 ± 1.5	0.004	<0.001
Lymphocytes (x10^4^/ml)	0.9 ± 0.1	5.8 ± 1.4	7.0 ± 2.1	4.0 ± 1.3	0.007	0.3
CD4+ T cells (x10^4^/ml)	0.4 ± 0.1	4.1 ± 1.0	5.1 ± 1.5	2.7 ± 1.0	<0.001	0.26
CD8+ T cells (x10^4^/ml)	0.3 ± 0.1	1.3 ± 0.3	1.7 ± 0.5	0.8 ± 0.2	0.02	0.18
CD4/CD8 ratio	1.8 ± 0.1	3.8 ± 0.4	4.1 ± 0.6	3.3 ± 0.4	<0.001	0.27

Data are presented as mean ± SEM. LD; Lymphocyte-dominant group, ND; Neutrophil-dominant group.

### HRCT scoring

Table 4 shows the HRCT scores evaluated in the lavaged segment. The scores for extent of bronchiectasis, bronchial wall thickening, and extent of multiple nodules or small nodules, in the ND group were significantly higher compared to the LD group (P < 0.05). The scores for the extent of sacculations/abscesses, and extent of mosaic perfusion in the ND group were also significantly higher in the ND group than the LD group (P < 0.05). Overall, the total lavaged segment scores in the ND group were significantly higher than the LD group (P < 0.001). On whole lung analysis, similar results were obtained (S5 Table). S3 and S4 Tables show the kappa value for each item of the CT score; kappa ranged from 0.466 to 1.00.

**Table 4:** HRCT scores of lavaged pulmonary segments.

	MAC patients			P value LD vs. ND
	All (N = 37)	LD (N = 22)	ND (N = 15)	
Severity of bronchiectasis	0.89 ± 0.11	0.73 ± 0.12	1.13 ± 0.19	0.06
Severity of bronchial wall thickening	0.73 ± 0.10	0.55 ± 0.11	1.00 ± 0.17	0.02
Extent of bronchiectasis	1.11 ± 0.15	0.82 ± 0.13	1.53 ± 0.28	0.016
Extent of multiple nodules or small nodules	1.16 ± 0.15	0.77 ± 0.10	1.73 ± 0.28	<0.001
Sacculations or abscesses	0.46 ± 0.11	0.18 ± 0.08	0.87 ± 0.20	<0.001
Extent of mosaic perfusion	0.08 ± 0.06	0 ± 0	0.27 ± 0.15	0.04
Collapse or consolidation	0.41 ± 0.09	0.32 ± 0.10	0.93 ± 0.15	0.001
Segment score	4.84 ± 0.57	3.27 ± 0.41	7.13 ± 1.02	<0.001

Data are presented by mean ± SEM. LD; Lymphocyte-dominant group, ND; Neutrophil-dominant group.

### Cytokine and chemokine analysis of BALF (Fig 2)

**Fig 2:**
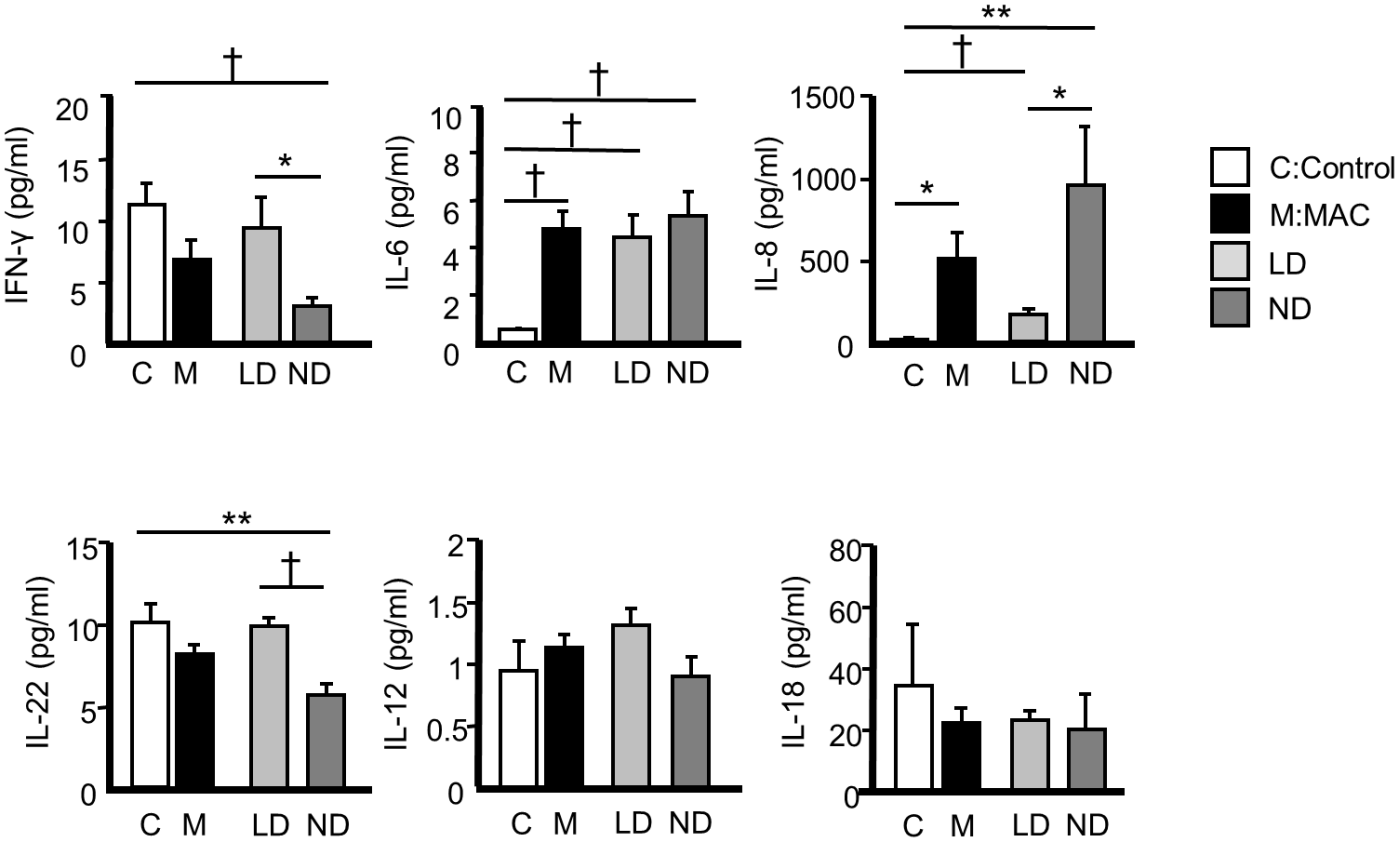
Cytokines and chemokines in bronchoalveolar lavage fluids of MAC patients. Data presented as means ± SEM. *P < 0.05 ** P < 0.01 † P < 0.001. LD: lymphocyte-dominant. ND: neutrophil-dominant. MAC: *Mycobacterium avium* complex.

On comparing the healthy subjects and patients with MAC infection, the levels of IL-6 and IL-8 were significantly higher (P<0.05). Levels of IFN-γ, IL-12, IL-18 and IL-22 were similar between the healthy subjects and MAC patients. Levels of IL-8 were significantly higher in the ND group compared to the LD group (P < 0.05). In contrast, levels of IFN-γ and IL-22 were significantly lower in the ND than the LD group (P < 0.05). The IL-6 levels were similar between the 2 groups. The IL-17A levels were under detectable in all subjects.

### Local cellular/immunological status and disease progression

Table 5 shows the patient characteristics of those for whom it was possible to evaluate the changes in HRCT findings without any treatment after the BAL (HRCT) procedure (N = 22). As described in the Materials and Methods section, 3 independent doctors subjectively evaluated the clinical course (stable or deteriorated) based on the HRCT changes. Subsequently, objective evaluation using the CT scores also showed that the initial evaluation corresponded to the subjective evaluation using HRCT for both lavaged-segment and whole lung scores (S6, S7 Tables).

**Table 5:** Characteristics of subjects who were followed-up without treatment after the bronchoalveolar lavage.

Characteristics	MAC patients			P value
	All (N = 22)	Stable* (N = 15)	Deteriorated† (N = 7)	Stable vs. Deterioration
Age(y), median[min-max]	67[45–78]	67[50–78]	57[45–76]	0.12
Sex (M/F)	4/18	2/13	2/5	0.78
Cigarette smoking status				
Current/Former/Never	0/4/18	0/2/13	0/2/5	0.78
MAC strain				
*M*. *avium*	20	13	7	0.95
*M*. *intracellulare*	2	2	0	

Definition of stable and deteriorated group were described in Material and Methods. Data are presented as the number of subjects except median age.

In the ND group, 83.3% (5/6) of subjects belonged to the deteriorated group, compared to only 12.5% (2/16) in the LD group (P < 0.01) (Table 6). The percentage of neutrophils was significantly higher in patients in the deteriorated group than the stable group (P = 0.04) (Table 6). Table 7 shows the comparison of the HRCT scores evaluated in the lavaged segment between the stable and deteriorated groups. Quite similar to the comparisons between the LD and ND groups, the scores for severity and extent of bronchiectasis, bronchial wall thickening, extent of multiple nodules or small nodules, and extent of sacculations/abscesses in the deteriorated group were significantly higher compared to the stable group (P < 0.05). Overall, the total lavaged-segment scores in the deteriorated group were significantly higher than the stable group (P < 0.01). On whole lung analysis, results similar to those of the lavaged segment were obtained (S8 Table).

**Table 6:** Local immunological status and disease progression after bronchoalveolar lavage in subjects who were followed-up without treatment.

	MAC patients		P value Stable vs. Deterioration
	Stable (N = 15)	Deteriorated (N = 7)	
Total cell count (x10^4^/ml)	26.4 ± 7.0	43.5 ± 25.3	0.39
Neutrophils (%)	7.4 ± 4.4	31.1 ± 13.2	0.04
Neutrophils (x10^4^/ml)	5.3 ± 4.5	29.6 ±22.1	0.15
Lymphocytes (%)	20.3 ± 4.3	17.9 ± 8.6	0.78
Lymphocytes (x10^4^/ml)	5.9 ± 2.0	3.5 ± 1.4	0.46
CD4+ T cells (x10^4^/ml)	4.4 ± 1.5	2.8 ± 1.2	0.49
CD8+ T cells (x10^4^/ml)	1.3 ± 0.5	0.6 ± 0.2	0.34
CD4/CD8 ratio	4.4 ± 0.8	4.6 ± 0.7	0.88
Number of subjects			
Lymphocyte-dominant (LD)	14	2	0.0043‡
Neutrophil-dominant (ND)	1	5	

Definition of stable and deteriorated group were described in Material and Methods. Date are presented by mean ± SEM.

‡Fisher’s exact test

**Table 7:** HRCT scores of the lavaged pulmonary segment at baseline who were followed-up without treatment after the bronchoalveolar lavage.

	MAC patients		P value
	Stable (N = 15)	Deteriorated (N = 7)	
Severity of bronchiectasis	0.67 ± 0.13	1.29 ± 0.29	0.03
Severity of bronchial wall thickening	0.47 ± 0.13	1.14 ± 0.26	0.02
Extent of bronchiectasis	0.67 ± 0.13	2.14 ± 0.34	<0.001
Extent of multiple nodules or small nodules	0.87 ± 0.10	1.71 ± 0.42	0.01
Sacculations or abscesses	0.27 ± 0.08	0.86 ± 0.20	0.03
Extent of mosaic perfusion	0.07 ± 0.07	0 ± 0	0.51
Collapse or consolidation	0.20 ± 0.15	0.43 ± 0.2	0.38
Segment score	3.2 ± 0.55	7.57 ± 1.45	0.003

Data are represented as mean ± SEM. LD; Lymphocyte-dominant group, ND; Neutrophil-dominant group, HRCT; high-resolution computed tomography.

S9 and S10 Tables show the comparisons of the HRCT scores evaluated in the lavaged segment and the whole lungs before and after the bronchoalveolar lavage in LD and ND group. The total lung score tended to be increased in ND group (P = 0.06, S10 Table). Levels of IL-8 were significantly higher in the deteriorated group compared to the stable group (P < 0.05). In contrast, levels of IL-22 were significantly lower in the deteriorated than the stable group (P < 0.05) (S11 Table).

## Discussion

In the present study, we demonstrated that ND immune response was associated with severe HRCT findings, not only in the lavaged pulmonary segments, but also in whole lungs, in patients with pulmonary MAC infection. The differences in the levels of several cytokines in the serum suggest the potentially distinct biological patterns of host reaction against MAC between the ND and LD groups. In addition, patients in the ND group showed greater progression on HRCT than those in the LD group.

The role of neutrophils in the impaired phagocytosis by macrophages, and thus, increased mycobacterial loads in the local lung tissue was suggested in previous studies [14,15]. In a murine model, a marked increase in neutrophil infiltration was observed in the lungs of genetically susceptible mice to mycobacterium infection [21]. In contrast, the role of lymphocytes in the early clearance of *Mycobacterium* mediated by poor adaptive immune response has been also suggested [16,17]. Collectively, these previous reports showing the contrasting roles of neutrophils and neutrophils suggest that the predominant immune response by either neutrophils or lymphocytes, as shown in the BAL cells in the present study, might determine the severity of radiographical findings in patients with pulmonary MAC infection.

Levels of IL-6 and IL-8 in the BALF were significantly higher in patients with MAC infection than in healthy patients, which is consistent with previous reports [13]. However, it is noteworthy that the IL-8 level increased only in the ND group, and not in the LD group, suggesting that IL-8 elevation is not a common feature in all patients with MAC infection. Given the chemoattractant role of neutrophils, IL-8 might play a significant role in ND inflammation, thereby showing association with severe HRCT findings. Similarly, significantly decreased levels of IFN-γ, associated with immune response by helper 1 T cells (Th1 cells), was observed only in the ND group. Thus, the lack of defense mechanisms against MAC infection associated with poor IFN-γ production might be involved in the enhancement of neutrophilic inflammation and radiographical severity [22,23].

A member of the IL-10 family, IL-22, is produced mainly by the T cells and natural-killer (NK) cells, and represents an effector cytokine of the Th17 lineage [24]. IL-17A and IL-22 have similar pathological effects in inflammatory diseases, such as psoriasis and arthritis [25,26]. Although the impact of IL-17A and IL-22 on the immune response to MAC infection is not clearly understood yet, in a murine model, both IL-17A and IL-22 were shown to contribute to granuloma formation by *M*. *tuberculosis*, which appears to have a protective function during mycobacterial infection [27]. Accordingly, decreased levels of IL-22 in the BALF in the ND group as shown in the current study suggest that the low IL-22 production might be associated with the neutrophilic response to MAC.

The cross-sectional nature of this study precludes the establishment of temporal relationships or causality inference. Thus, it cannot be ruled out that the increase in neutrophils seen might be the result of the advanced disease itself. Thus, we selected 22 patients who had been followed-up without any treatment after BAL (HRCT) procedure and classified them into stable and deteriorated groups (see Materials and methods for a detailed definition). We demonstrated that patients in the ND group showed a significantly higher frequency in the deteriorated group (83.3%) than the LD group (12.5%). The neutrophil count and serum IL-8 were significantly higher in the deteriorated group than in the stable group, whereas IL-22 was lower in the deteriorated group. In addition, the baseline HRCT scores, particularly the extent of multiple nodules or small nodules, were significantly higher in the deteriorated group than the stable group. Accordingly, the type of cellular response may predispose a patient to MAC infection and patients who exhibit a predominantly neutrophilic response are associated with deterioration of the disease.

The present study confirmed the results from previous studies, showing that the CD4/CD8 ratio in the BALF was elevated in patients with pulmonary MAC infection. An elevation of the CD4/CD8 ratio in the BALF is a well-known feature of sarcoidosis, which is characterized by granuloma formation associated with an increased number of activated T cells [28,29]. Given the similar radiographic findings, i.e., centrilobular nodular lesions, and the high prevalence of middle-aged women among those with both sarcoidosis and pulmonary MAC infection, MAC infection should be included in the differential diagnosis of patients with increased CD4^+^ T cells and CD4/CD8 ratio in the BALF.

This study has several significant limitations. First, in our department, BAL was conducted for patients with suspected NTM infection, only when the sputum cultures failed to demonstrate a positive result several times. Thus, despite our effort to recruit patients who needed BAL for diagnosis, the sample size was small. Second, the classification of patients based on the predominant BALF cell type (ND and LD patients) was adopted due to a similar percentage level and differentiation, and its clinical convenience. It is uncertain whether this classification is clinically relevant. However, this classification showed significant differences with regard to radiographical severity, several cytokine levels, and disease progression, suggesting the potential for clinical utility. Third, we did not measure albumin and/or urea in the BALF in our samples, considering the dilatation rate of BALF. However, as mentioned in the Material and Methods section, our BAL procedure was almost identical for all subjects. Thus, we believe that the difference of the dilution rates in the BALF was minimal and did not influence our cytokine level results. Lastly, this study considered patients who underwent BAL; thus, it might not be possible to extrapolate it to all patients with pulmonary MAC infection. Analyses using more non-invasive methods, such as sputum samples, need to be done for clinical relevance. However, the results showing the differences in HRCT findings between the stable and deteriorated groups (as shown in Table 7 and S6 Table) may provide clinically useful information on the prediction of future disease deterioration, and the timing and patient selection for treatment.

In conclusion, our study provides evidence that the type of local cellular immune response in the lung correlated with the radiographical severity and biological difference in patients with pulmonary MAC infection. Understanding the precise mechanisms that underlie the different cellular responses to MAC infection may lead to further insights regarding its pathogenesis, presentation, and treatment.

## Supporting information

S1 Table Summary of the HRCT scoring system of the whole lung. BPs; bronchopulmonary segments.
(PDF)

S2 Table Peripheral white blood cell differentiation of the groups. Data are presented as mean ± SEM. *Of 22 subjects, 16 samples were available for peripheral blood analysis. LD; Lymphocyte-dominant group, ND; Neutrophil-dominant group.
(PDF)

S3 Table Cohen’s Kappa values for HRCT scores of lavaged pulmonary segments. (PDF)

S4 Table Cohen’s kappa values for HRCT scores of the whole lung. (PDF)

S5 Table HRCT score of the whole lung. Data are presented by mean ± SEM. LD; Lymphocyte-dominant group, ND; Neutrophil-dominant group.
(PDF)

S6 Table Comparisons of HRCT scores of the lavaged pulmonary segment in subjects who were followed-up without treatment before and after the bronchoalveolar lavage (in Stable and Deteriorated group). Data are presented by mean ± SEM.
(PDF)

S7 Table Comparisons of HRCT scores of the whole lungs in subjects who were followed-up without treatment before and after the bronchoalveolar lavage (in Stable and Deteriorated group). Data are presented by mean ± SEM.
(PDF)

S8 Table HRCT score of the whole lungs at baseline who had been followed-up without treatment after the bronchoalveolar lavage. Data are represented as mean ± SEM. LD; Lymphocyte-dominant group, ND; MAC, Mycobacterium avium complex; Neutrophil-dominant group, HRCT; high-resolution computed tomography.
(PDF)

S9 Table Comparisons of HRCT scores of the lavaged pulmonary segment in subjects who were followed-up without treatment before and after the bronchoalveolar lavage (in LD and ND group). Data are presented by mean ± SEM.
(PDF)

S10 Table Comparisons of HRCT scores of the whole lungs in subjects who were followed-up without treatment before and after the bronchoalveolar lavage (in LD and ND group). Data are presented by mean ± SEM.
(PDF)

S11 Table Cytokine levels in the bronchoalveolar lavage fluid of MAC patients who were followed-up without treatment after the lavage. Data are presented by mean ± SEM.
(PDF)

S1 Fig Representative HRCT images. (TIF)

S1 File Dataset Control and Mac Pt. (XLS)
