# Supplementary material for: Neutrophil predominance in bronchoalveolar lavage fluid is associated with disease severity and progression of HRCT findings in pulmonary Mycobacterium avium infection
Source: PLoS One. 2018 Feb 5;13(2):e0190189. doi: 10.1371/journal.pone.0190189 (PMC5798761; doi:10.1371/journal.pone.0190189)
Supplement: S1 Table — BPs; bronchopulmonary segments. (PDF) [file pone.0190189.s001.pdf]

S1 Table. Summary of the HRCT scoring system of the whole lung

Category	Score			
	0	1	2	3
Severity of Bronchiectasis	Absent	Mild (1-2 x diameter of adjacent vessel)	Moderate (2-3 x diameter of adjacent vessel)	Severe (>3 x diameter of adjacent vessel)
Severity of bronchial wall thickening	Absent	Mild (<0.5 x diameter of adjacent vessel)	Moderate (0.5-1 x diameter of adjacent vessel)	Severe (>1 x diameter of adjacent vessel)
Extent of bronchiectasis (No. of BPs)	Absent	1-5	6-9	>9
Extent of multiple nodules or small nodules (No. of BPs)	Absent	1-5	6-9	>9
Sacculations or abscesses (No. of BPs)	Absent	1-5	6-9	>9
Extent of mosaic perfusion (No. of BPs)	Absent	1-5	6-9	>9
Collapse or consolidation (No. of BPs)	Absent	1-3	4-6	>6

BPs; bronchopulmonary segments.
