# Supplementary material for: Neutrophil predominance in bronchoalveolar lavage fluid is associated with disease severity and progression of HRCT findings in pulmonary Mycobacterium avium infection
Source: PLoS One. 2018 Feb 5;13(2):e0190189. doi: 10.1371/journal.pone.0190189 (PMC5798761; doi:10.1371/journal.pone.0190189)
Supplement: S2 Table — Data are presented as mean ± SEM. *Of 22 subjects, 16 samples were available for peripheral blood analysis. LD; Lymphocyte-dominant group, ND; Neutrophil-dominant group. (PDF) [file pone.0190189.s002.pdf]

S2 Table. Peripheral white blood cell differentiation of the groups

	Control (N=16*)	MAC patients			P value	
		All (N=37)	LD (N=22)	ND (N=15)	Control vs. MAC	LD vs. ND
White blood cells (x10^4/ml)	5506 ± 317	5208 ± 178	5159 ± 256	5280 ± 236	0.39	0.74
Neutrophils (x10^4/ml)	3250 ± 228	3381 ± 146	3325 ± 204	3462 ± 208	0.63	0.65
Lymphocytes (x10^4/ml)	1625 ± 121	1421 ± 77	1424 ± 112	1416 ± 103	0.16	0.96
Monocytes (x10^4/ml)	394 ± 42	288 ± 19	281 ± 26	295 ± 30	0.01	0.73
Eosinophils (x10^4/ml)	219 ± 40	91 ± 14	104 ± 19	71 ± 17	<0.001	0.24

Data are presented as mean ± SEM.

*Of 22 subjects, 16 samples were available for peripheral blood analysis

LD; Lymphocyte-dominant group, ND; Neutrophil-dominant group.
