# Supplementary material for: Neutrophil predominance in bronchoalveolar lavage fluid is associated with disease severity and progression of HRCT findings in pulmonary Mycobacterium avium infection
Source: PLoS One. 2018 Feb 5;13(2):e0190189. doi: 10.1371/journal.pone.0190189 (PMC5798761; doi:10.1371/journal.pone.0190189)
Supplement: S3 Table — (PDF) [file pone.0190189.s003.pdf]

S3 Table. Cohen's Kappa values for HRCT scores of lavaged pulmonary segments

	Kappa value	95%CI
Severity of bronchiectasis	0.532	0.288 - 0.776
Severity of bronchial wall thickening	0.466	0.237 – 0.695
Extent of bronchiectasis	0.614	0.402 – 0.826
Extent of multiple nodules or small nodules	0.806	0.627 – 0.985
Sacculations or abscesses	0.896	0.758 – 1.034
Extent of mosaic perfusion	1.000	—
Collapse or consolidation	0.944	0.839 – 1.049
