# Supplementary material for: Neutrophil predominance in bronchoalveolar lavage fluid is associated with disease severity and progression of HRCT findings in pulmonary Mycobacterium avium infection
Source: PLoS One. 2018 Feb 5;13(2):e0190189. doi: 10.1371/journal.pone.0190189 (PMC5798761; doi:10.1371/journal.pone.0190189)
Supplement: S4 Table — (PDF) [file pone.0190189.s004.pdf]

S4 Table. Cohen’s kappa values for HRCT scores of the whole lung

	Kappa value	95%CI
Severity of bronchiectasis	0.506	0.269 - 0.744
Severity of bronchial wall thickening	0.515	0.256 – 0.775
Extent of bronchiectasis	0.786	0.631 – 0.941
Extent of multiple nodules or small nodules	0.754	0.568 – 0.941
Sacculations or abscesses	0.705	0.491 – 0.918
Extent of mosaic perfusion	0.789	0.390 – 1.189
Collapse or consolidation	0.624	0.385 – 0.862
