# Supplementary material for: Neutrophil predominance in bronchoalveolar lavage fluid is associated with disease severity and progression of HRCT findings in pulmonary Mycobacterium avium infection
Source: PLoS One. 2018 Feb 5;13(2):e0190189. doi: 10.1371/journal.pone.0190189 (PMC5798761; doi:10.1371/journal.pone.0190189)
Supplement: S5 Table — Data are presented by mean ± SEM. LD; Lymphocyte-dominant group, ND; Neutrophil-dominant group. (PDF) [file pone.0190189.s005.pdf]

S5 Table. HRCT score of the whole lung

	MAC patients			P value LD vs. ND
	All (N=37)	LD (N=22)	ND (N=15)	
Severity of bronchiectasis	0.87 ± 0.63	0.55 ± 0.11	1.33 ± 0.49	<0.001
Severity of bronchial wall thickening	0.70 ± 0.09	0.46 ± 0.11	1.07 ± 0.07	<0.001
Extent of bronchiectasis	0.90 ± 0.09	0.55 ± 0.11	1.40 ± 0.16	<0.001
Extent of multiple nodules or small nodules	1.49 ± 0.12	1.05 ± 0.12	2.13 ± 0.24	<0.001
Sacculations or abscesses	0.70 ± 0.12	0.36 ± 0.11	1.20 ± 0.17	<0.001
Extent of mosaic perfusion	0.11 ± 0.07	0 ± 0	0.27 ± 0.15	0.04
Collapse or consolidation	0.57 ±0.10	0.32 ± 0.10	0.93 ± 0.15	0.001
Total lung score	5.32 ± 0.55	3.27 ± 0.44	8.33 ± 0.65	<0.001

Data are presented by mean ± SEM.

LD; Lymphocyte-dominant group, ND; Neutrophil-dominant group.
