# Supplementary material for: Neutrophil predominance in bronchoalveolar lavage fluid is associated with disease severity and progression of HRCT findings in pulmonary Mycobacterium avium infection
Source: PLoS One. 2018 Feb 5;13(2):e0190189. doi: 10.1371/journal.pone.0190189 (PMC5798761; doi:10.1371/journal.pone.0190189)
Supplement: S6 Table — Data are presented by mean ± SEM. (PDF) [file pone.0190189.s006.pdf]

S6 Table. Comparisons of HRCT scores of the lavaged pulmonary segment in subjects who were followed-up without treatment before and after the bronchoalveolar lavage (in Stable and Deteriorated group)

	Stable (N=15)		P value	Deteriorated (N=7)		P value
	before	after		before	after	
Severity of bronchiectasis	0.67 ± 0.13	0.67 ± 0.13	n.s.	1.29 ± 0.29	1.57 ± 0.37	0.17
Severity of bronchial wall thickening	0.47 ± 0.13	0.47 ± 0.13	n.s.	1.14 ± 0.26	1.14 ± 0.26	n.s.
Extent of bronchiectasis	0.67 ± 0.13	0.67 ± 0.13	n.s.	2.14 ± 0.34	2.29 ± 0.29	0.36
Extent of multiple nodules or small nodules	0.87 ± 0.1	0.87 ± 0.1	n.s.	1.71 ± 0.42	2.14 ± 0.34	0.20
Sacculations or abscesses	0.27 ± 0.12	0.27 ± 0.12	n.s.	0.86 ± 0.26	1.14 ± 0.34	0.17
Extent of mosaic perfusion	0.07 ± 0.07	0.07 ± 0.07	n.s.	0.0 ± 0.0	0.0 ± 0.0	n.s.
Collapse or consolidation	0.2 ± 0.15	0.2 ± 0.15	n.s.	0.43 ± 0.2	0.71 ± 0.29	0.36
Segment score	3.2 ± 0.55	3.2 ± 0.55	n.s.	7.57 ± 1.45	9.0 ± 1.23	0.06

Data are presented by mean ± SEM.
