# Supplementary material for: Neutrophil predominance in bronchoalveolar lavage fluid is associated with disease severity and progression of HRCT findings in pulmonary Mycobacterium avium infection
Source: PLoS One. 2018 Feb 5;13(2):e0190189. doi: 10.1371/journal.pone.0190189 (PMC5798761; doi:10.1371/journal.pone.0190189)
Supplement: S7 Table — Data are presented by mean ± SEM. (PDF) [file pone.0190189.s007.pdf]

S7 Table. Comparisons of HRCT scores of the whole lungs in subjects who were followed-up without treatment before and after the bronchoalveolar lavage (in Stable and Deteriorated group)

	Stable (N=15)		P value	Deteriorated (N=7)		P value
	before	after		before	after	
Severity of bronchiectasis	0.47 ± 0.13	0.47 ± 0.13	n.s.	1.14 ± 0.26	1.57 ± 0.2	0.08
Severity of bronchial wall thickening	0.33 ± 0.13	0.33 ± 0.13	n.s.	0.86 ± 0.14	1.0 ± 0.0	0.36
Extent of bronchiectasis	0.47 ± 0.13	0.47 ± 0.13	n.s.	1.14 ± 0.26	1.29 ± 0.18	0.36
Extent of multiple nodules or small nodules	1.0 ± 0.1	1.0 ± 0.1	0.16	2.14 ± 0.34	2.57 ± 0.3	0.045
Sacculations or abscesses	0.4 ± 0.13	0.4 ± 0.13	0.33	1.0 ± 0.22	1.57 ± 0.2	0.047
Extent of mosaic perfusion	0.07 ± 0.07	0.07 ± 0.07	n.s.	0.14 ± 0.14	0.14 ± 0.14	n.s.
Collapse or consolidation	0.33 ± 0.16	0.27 ± 0.12	0.33	0.43 ± 0.2	0.71 ± 0.29	0.17
Total Lung score	3.07 ± 0.57	3.0 ± 0.53	0.33	6.86 ± 1.16	8.86 ± 0.77	0.01

Data are presented by mean ± SEM.
