# Supplementary material for: Neutrophil predominance in bronchoalveolar lavage fluid is associated with disease severity and progression of HRCT findings in pulmonary Mycobacterium avium infection
Source: PLoS One. 2018 Feb 5;13(2):e0190189. doi: 10.1371/journal.pone.0190189 (PMC5798761; doi:10.1371/journal.pone.0190189)
Supplement: S8 Table — Data are represented as mean ± SEM. LD; Lymphocyte-dominant group, ND; MAC, Mycobacterium avium complex; Neutrophil-dominant group, HRCT; high-resolution computed tomography. (PDF) [file pone.0190189.s008.pdf]

S8 Table. HRCT score of the whole lungs at baseline who had been followed-up without treatment after the bronchoalveolar lavage

	MAC patients		P value
	Stable (N=15)	Deteriorated (N=7)	
Severity of bronchiectasis	0.47 ± 0.13	1.14 ± 0.26	0.02
Severity of bronchial wall thickening	0.33 ± 0.13	0.86 ± 0.14	0.02
Extent of bronchiectasis	0.47 ± 0.13	1.14 ± 0.34	0.02
Extent of multiple nodules or small nodules	1.0 ± 0.1	2.14 ± 0.34	<0.001
Sacculations or abscesses	0.4 ± 0.13	1.0 ± 0.22	0.02
Extent of mosaic perfusion	0.07 ± 0.07	0.14 ± 0.14	0.58
Collapse or consolidation	0.33 ± 0.16	0.43 ± 0.2	0.73
Segment score	3.07 ± 0.57	6.86 ± 1.16	0.003

Data are represented as mean ± SEM.

LD; Lymphocyte-dominant group, ND; MAC, Mycobacterium avium complex; Neutrophil-dominant group, HRCT; high-resolution computed tomography.
