# Supplementary material for: Neutrophil predominance in bronchoalveolar lavage fluid is associated with disease severity and progression of HRCT findings in pulmonary Mycobacterium avium infection
Source: PLoS One. 2018 Feb 5;13(2):e0190189. doi: 10.1371/journal.pone.0190189 (PMC5798761; doi:10.1371/journal.pone.0190189)
Supplement: S9 Table — Data are presented by mean ± SEM. (PDF) [file pone.0190189.s009.pdf]

S9 Table. Comparisons of HRCT scores of the lavaged pulmonary segment in subjects who were followed-up without treatment before and after the bronchoalveolar lavage (in LD and ND group)

	LD group (N=16)		P value	ND group (N=6)		P value
	before	after		before	after	
Severity of bronchiectasis	0.62 ± 0.13	0.62 ± 0.13	n.s.	1.5 ± 0.22	1.83 ± 0.31	0.17
Severity of bronchial wall thickening	0.44 ± 0.13	0.44 ± 0.13	n.s.	1.33 ± 0.21	1.33 ± 0.21	n.s.
Extent of bronchiectasis	0.75 ± 0.15	0.75 ± 0.15	n.s.	2.17 ± 0.4	2.33 ± 0.33	0.36
Extent of multiple nodules or small nodules	0.81 ± 0.1	0.94 ± 0.11	0.33	2.0 ± 0.37	2.17 ± 0.4	0.36
Sacculations or abscesses	0.19 ± 0.1	0.25 ± 0.11	0.33	1.17 ± 0.17	1.33 ± 0.33	0.36
Extent of mosaic perfusion	0.0 ± 0.0	0.0 ± 0.0	n.s.	0.17 ± 0.17	0.17 ± 0.17	n.s.
Collapse or consolidation	0.06 ± 0.06	0.06 ± 0.06	n.s.	0.83 ± 0.31	1.17 ± 0.31	0.36
Segment score	2.88 ± 0.42	3.06 ± 0.43	0.33	9.17 ± 0.98	10.33 ± 0.67	0.13

Data are presented by mean ± SEM.
