# Supplementary material for: Neutrophil predominance in bronchoalveolar lavage fluid is associated with disease severity and progression of HRCT findings in pulmonary Mycobacterium avium infection
Source: PLoS One. 2018 Feb 5;13(2):e0190189. doi: 10.1371/journal.pone.0190189 (PMC5798761; doi:10.1371/journal.pone.0190189)
Supplement: S10 Table — Data are presented by mean ± SEM. (PDF) [file pone.0190189.s010.pdf]

S10 Table. Comparisons of HRCT scores of the whole lungs in subjects who were followed-up without treatment before and after the bronchoalveolar lavage (in LD and ND group)

	LD group (N=16)		P value	ND group (N=6)		P value
	before	after		before	after	
Severity of bronchiectasis	0.44 ± 0.13	0.5 ± 0.13	0.33	1.33 ± 0.21	1.67 ± 0.21	0.17
Severity of bronchial wall thickening	0.31 ± 0.12	0.38 ± 0.13	0.33	1.0 ± 0.0	1.0 ± 0.0	n.s.
Extent of bronchiectasis	0.44 ± 0.13	0.5 ± 0.13	0.33	1.33 ± 0.21	1.33 ± 0.21	n.s.
Extent of multiple nodules or small nodules	1.13 ± 0.16	1.19 ± 0.16	0.33	2.0 ± 0.37	2.33 ± 0.42	0.17
Sacculations or abscesses	0.38 ± 0.13	0.5 ± 0.16	0.16	1.17 ± 0.17	1.5 ± 0.22	0.17
Extent of mosaic perfusion	0.0 ± 0.0	0.0 ± 0.0	n.s.	0.33 ± 0.21	0.33 ± 0.21	n.s.
Collapse or consolidation	0.19 ± 0.1	0.19 ± 0.1	n.s.	0.83 ± 0.31	1.0 ± 0.26	0.61
Total Lung score	2.89 ± 0.51	3.25 ± 0.56	0.25	8.0 ± 0.73	9.17 ± 0.79	0.06

Data are presented by mean ± SEM.
