# Supplementary material for: Neutrophil predominance in bronchoalveolar lavage fluid is associated with disease severity and progression of HRCT findings in pulmonary Mycobacterium avium infection
Source: PLoS One. 2018 Feb 5;13(2):e0190189. doi: 10.1371/journal.pone.0190189 (PMC5798761; doi:10.1371/journal.pone.0190189)
Supplement: S11 Table — Data are presented by mean ± SEM. (PDF) [file pone.0190189.s011.pdf]

S11 Table.Cytokine levels in the bronchoalveolar lavage fluid of MAC patients who were followed-up without treatment after the lavage

	MAC patients		P value Stable vs. Deterioration
	Stable (N=15)	Deteriorated (N=7)	
IL-6 (pg/ml)	4.0 ± 1.0	3.9 ± 1.2	0.94
IL-8 (pg/ml)	127.3 ± 32.8	1163.4 ± 719.2	0.04
IL-12 (pg/ml)	1.4 ± 0.2	1.1 ± 0.2	0.43
IFN-gamma (pg/ml)	8.4 ± 3.2	5.3 ± 2.2	0.53
IL-22 (pg/ml)	9.3 ± 0.9	6.0 ± 1.0	0.04
IL-18 (pg/ml)	21.5 ± 3.8	34.5 ± 22.9	0.43
